# Growth Kinetics of *Listeria monocytogenes* and *Salmonella enterica* on Dehydrated Vegetables during Rehydration and Subsequent Storage

**DOI:** 10.3390/foods12132561

**Published:** 2023-06-30

**Authors:** Megan L. Fay, Joelle K. Salazar, Yuying Ren, Zihui Wu, Madhuri Mate, Bashayer A. Khouja, Pravalika Lingareddygari, Girvin Liggans

**Affiliations:** 1 Division of Food Processing Science and Technology, U. S. Food and Drug Administration, Bedford Park, IL 60501, USA; megan.fay@fda.hhs.gov (M.L.F.); bashayer.khouja@fda.hhs.gov (B.A.K.);; 2 Department of Food Science and Nutrition, Illinois Institute of Technology, Bedford Park, IL 60501, USA; 3 Office of Food Safety, U. S. Food and Drug Administration, College Park, MD 20740, USA; girvin.liggans@fda.hhs.gov

**Keywords:** dried produce, foodborne pathogens, growth rates, predictive modeling

## Abstract

Dehydrated vegetables have low water activities and do not support the proliferation of pathogenic bacteria. Once rehydrated, vegetables can be incorporated into other foods or held for later use. The aim of this study was to examine the survival and proliferation of *Listeria monocytogenes* and *Salmonella enterica* on dehydrated vegetables during rehydration and subsequent storage. Carrots, corn, onion, bell peppers, and potatoes were heat dehydrated, inoculated at 4 log CFU/g, and rehydrated at either 5 or 25 °C for 24 h. Following rehydration, vegetables were stored at 5, 10, or 25 °C for 7 d. Both *L. monocytogenes* and *S. enterica* survived on all vegetables under all conditions examined. After 24 h of rehydration at 5 °C, pathogen populations on the vegetables were generally <1.70 log CFU/g, whereas rehydration at 25 °C resulted in populations of 2.28 to 6.25 log CFU/g. The highest growth rates during storage were observed by *L. monocytogenes* on potatoes and *S. enterica* on carrots (2.37 ± 0.61 and 1.63 ± 0.18 log CFU/g/d, respectively) at 25 °C when rehydration occurred at 5 °C. Results indicate that pathogen proliferation on the vegetables is both rehydration temperature and matrix dependent and highlight the importance of holding rehydrated vegetables at refrigeration temperatures to hinder pathogen proliferation. Results from this study inform time and temperature controls for the safety of these food products.

## 1. Introduction

Plant foods, including vegetables, are popular around the world because of their nutritional value and abundance. However, the high moisture content and high water activity (a_w_) of these food commodities translates to a short shelf life. Various techniques are therefore employed to preserve these foods for longer periods, including desiccation methods. Dehydration using heat is a common method to desiccate vegetables, resulting in the reduction of a_w_ (to <0.70) and also reducing the weight, which is convenient for both transportation and storage. To minimize any organoleptic changes of the vegetables during dehydration, treatments are usually mild and use low heat (<80 °C) [1]. Vegetables are often heat dehydrated using a tunnel of hot air, where the internal product temperature does not often exceed 35–45 °C [2]. Therefore, heat dehydration is generally not an effective method for reducing the microbial load or for the inactivation of foodborne bacterial pathogens, including *Listeria monocytogenes* and *Salmonella enterica*.

While dehydrated foods, including vegetables, do not support the growth of foodborne pathogens, *L. monocytogenes* and *S. enterica* are known to persist long-term in low a_w_ (<0.70) foods including nuts and seeds [3,4,5,6,7], spices [4,8,9], dried fruits [10,11], powdered milk products [6,12], powdered infant formula [13], flour [14,15], and confectionaries [7,16,17]. For example, *S. enterica*, *E. coli* O157:H7, and *L. monocytogenes* have been documented to survive for 1 year on walnut kernels when stored at −20, 4, and 23 °C [3] and on peanut and pecan kernels when stored at −24, 4, and 22 °C [5]. On dried fruits, *S. enterica* survived for 182 days on dried strawberries and for 242 days on dried cranberries and raisins stored at 4 °C [10]. In another study, *S. enterica*, *Escherichia coli* O157:H7, and *L. monocytogenes* survived for 180 days on dried dates, tomatoes, and pluots stored at ambient temperature and refrigerated [11]. All pathogens were documented to survive at higher populations on dates (pH 5.59–5.83) than on tomatoes (pH 3.80) or pluots (pH 3.45), indicating that the pH of the low a_w_ food matrix plays a role in survival. While no published studies have examined the survival of foodborne pathogens on dehydrated vegetables, their relatively neutral pH may allow for long-term persistence.

Foodborne outbreaks in the U.S. and internationally due to *S. enterica*, Shiga toxin-producing *E. coli*, or *Bacillus cereus* contamination of dried fruit [18], dried fungi [19,20], and spices made from dried herbs and vegetables [21,22,23,24] have also occurred. In one outbreak, a spice mix containing dried vegetables (including carrots, onions, and parsnips) caused 174 salmonellosis cases in Sweden in 2015 [21]. The implicated spice mix was used at a restaurant and incorporated into foods with a higher final a_w_. While no reported outbreaks of *L. monocytogenes* have been associated with low a_w_ foods, there have been several recalls of various dry food products due to possible *L. monocytogenes* contamination, including granola [25], walnuts [26], and dried apricots [27].

Dehydrated vegetables are typically prepared for consumption by either direct incorporation into other foods with higher a_w_, such as soups, deli salads, and sandwiches, or rehydrated prior to incorporation/consumption. In either case, rehydration leads to an increase in the a_w_ and moisture content. Rehydrated vegetables can also be stored for later use at various temperatures. Combined with the relatively neutral pH of vegetables, the increase in a_w_ (to >0.92) and moisture creates an environment conducive to the proliferation of vegetative bacteria. Therefore, product assessments are needed to determine the extent to which rehydrated vegetables support the proliferation of foodborne pathogens. Studies have also shown that the water temperature used for rehydration impacts the rehydration kinetics of plant foods, including vegetables and fruits [28,29,30], which may also affect the survival or proliferation of foodborne pathogens.

The FDA Food Code lists cut leafy greens, cut melons, and cut tomatoes as foods requiring time and temperature control for safety (TCS foods) [31]. However, there are no time and temperature guidelines for safety for the rehydration and storage of cut, previously dehydrated vegetables. It is unclear if the population dynamics of bacterial pathogens are similar or different on dehydrated vegetables once rehydrated and stored compared to their fresh counterparts. Therefore, the objective of this study was to examine the survival and proliferation of *L. monocytogenes* and *S. enterica* on dehydrated vegetables during rehydration and subsequent storage. Results from this study will aid in understanding the time and temperature combinations for rehydration and subsequent storage for these food products which support the proliferation of *L. monocytogenes* and *S. enterica*.

## 2. Materials and Methods

### 2.1. Vegetable Preparation and Dehydration

Sweet corn on the cob, carrots, yellow onions, green bell peppers, and Yukon Gold potatoes were sourced from local retail grocers (Chicago, IL, USA). Corn was husked and the kernels were cut off the cob. Carrots, onions, and potatoes were peeled and peppers were deseeded. Carrots, onions, potatoes, and peppers were chopped using a commercial food chopper (DC12 ½” Heavy-duty Vegetable Dicer, Garde, Chicago, IL, USA). Chopped carrots and potatoes measured 1.3 × 1.3 cm, while onions and peppers measured 1.3 × 0.3 cm. Vegetables were arranged on metal racks with wax paper and dehydrated at 60 °C for 24 h using a commercial food dehydrator (Excalibur model EXC10EL, The Legacy Companies, Weston, FL, USA). The pH, water activity (a_w_), and moisture contents of triplicate samples of fresh vegetables and the vegetables during dehydration (after 2, 4, 6, 8, 12, 16, and 24 h) were measured (see Section 2.6).

### 2.2. Strains, Culture Conditions, and Inoculum Preparation

Four-strain cocktails of either *Salmonella enterica* or *Listeria monocytogenes* were used in this study. The *S. enterica* strains used were Enteritidis PT30 (ATCC BAA-1045), Agona 447967 (roasted oats cereal isolate), Typhimurium 46249 (cantaloupe outbreak isolate), and Newport 36796 (CFSAN046260, tomato outbreak isolate). The *L. monocytogenes* strains were ScottA (clinical isolate), LS806 (isolated from hummus), LS3132 (isolated from avocado), and LS1863 (FDA1142659-C001-001, enoki mushroom outbreak isolate). All *L. monocytogenes* strains were resistant to rifampicin (100 µg/mL).

Strains were cultured in Tryptic Soy Broth (TSB; Becton, Dickinson and Co., Sparks, MD, USA) for 16–18 h at 37 °C and then washed twice with Butterfields’s Phosphate Buffer (BPB, pH 7.2). Equal volumes of each washed culture were combined to create a four-strain cocktail of either *S. enterica* or *L. monocytogenes* (ca. 9 log CFU/mL). To verify population levels, the cocktails were serially diluted in BPB and plated onto Tryptic Soy Agar (TSA; Becton, Dickinson and Co., Sparks, MD, USA). Agar plates were incubated for 24–48 h at 37 °C prior to enumeration.

### 2.3. Inoculation of Dehydrated Vegetables

Dehydrated vegetables (400 g) were transferred to 3-L stomacher bags and inoculated with either the *L. monocytogenes* or *S. enterica* cocktail at 4 log CFU/g. The stomacher bags were hand shaken for 5 min to uniformly distribute the inoculum. Inoculated vegetables were arranged onto foil pans and dried at ambient temperature for 24 h in a biosafety cabinet with the blower on. After 24 h, triplicate 10-g samples were used for pathogen enumeration (see Section 2.7).

### 2.4. Rehydration of Dehydrated Vegetables

After 24 h of drying, the inoculated dehydrated vegetables were rehydrated 1:10 in water in an 8 L metal bowl at either 5 or 25 °C for 24 h. Both the water and air temperature were maintained at either 5 or 25 °C. During rehydration, vegetable samples were removed from the water at 2.5, 5, and 30 min, and 1, 2, 4, 6, and 24 h. At each of these timepoints, the vegetables were stirred and approximately 50 g were removed from the water and strained for 10 min using a 10 cm diameter strainer. Triplicate 10 g samples were used for pathogen enumeration (see Section 2.7) and duplicate 10 g samples were used for moisture content analysis (see Section 2.6). After 24 h of rehydration, the remaining vegetables were strained for 10 min using an 8 L colander.

### 2.5. Storage of Rehydrated Vegetables

The remaining strained rehydrated vegetables were portioned into 8 oz deli containers with lids (40 g each). Deli containers were stored at 5, 10, or 25 °C for 7 d. After 0, 1, 3, 5, and 7 d, pathogens were enumerated from the samples (see Section 2.7). Triplicate independent trials were conducted for each vegetable, temperature, and pathogen combination (dehydration through storage).

### 2.6. Measurement of pH, Water Activity (a_w_), and Moisture Content

For pH, a 10 g sample of vegetable was stomached for 1 min with 10 mL of distilled autoclaved water in a stomacher (400 Circulator Lab Blender, Seward, UK) and the pH of the homogenate was measured with a pH meter (MP220 pH meter, Mettler Toledo, Columbus, OH, USA). To measure a_w_, a 1 g sample of vegetable was measured using an a_w_ meter (Aqualab 4TE, Meter Group, Pullman, WA, USA). For moisture content, a 10 g sample of vegetable was placed into an oven at 100 °C for 24 h. The solid weight after 24 h was measured and the moisture content was then calculated.

### 2.7. Enumeration of L. monocytogenes and S. enterica

Vegetable samples (10 g) were homogenized in a stomacher at 1:10 with either Buffered *Listeria* Enrichment Broth (BLEB; Becton, Dickinson and Co., Sparks, MD, USA) or BPB for *L. monocytogenes* or *S. enterica*, respectively. Homogenates were serially diluted and plated onto Brain Heart Infusion Agar (BHIA; Becton, Dickinson and Co., Sparks, MD, USA) with rifampicin (BHIA^rif^) or onto TSAYE with Xylose Lysine Deoxycholate (XLD; Becton, Dickinson and Co., Sparks, MD, USA) agar overlay for enumeration of *L. monocytogenes* or *S. enterica*, respectively. Agar plates were incubated at 37 °C for 24–48 h. Data were expressed as log CFU/g. The limit of enumeration of the plate count assay was 1.70 log CFU/g.

### 2.8. Modeling of Growth Kinetics and Statistical Analysis

Growth kinetics (i.e., growth rates and lag phases) of both *L. monocytogenes* and *S. enterica* during 7 d storage at 5, 10, or 25 °C were determined using the DMFit v 3.0 add-in for Excel (Baranyi and Roberts, 1994). Differences between growth rates were statistically analyzed using ANCOVA with Tukey’s post hoc test (α = 0.05). Differences in pH, a_w_, moisture contents, and populations were statistically analyzed using ANOVA with Tukey’s post hoc test (α = 0.05).

## 3. Results

### 3.1. Characteristics of the Fresh, Dehydrated, and Rehydrated Vegetables

The pH and a_w_ values of the fresh vegetables used in this study are presented in Table 1. The pH values of the fresh vegetables ranged from 6.15 ± 0.06 (for bell pepper) to 7.00 ± 0.10 (for carrot). The pH of the bell pepper was significantly lower than all the other vegetables. The a_w_ of all the fresh vegetables ranged from 0.951 ± 0.015 (for corn) to 0.960 ± 0.008 (for potato). There was no significant difference in the a_w_ values between vegetables. After dehydration of the vegetables at 60 °C for 24 h, the pH values ranged from 5.73 ± 0.07 (for onion) to 6.77 ± 0.02 (for corn). When comparing the pH values of the five vegetables after dehydration, all were significantly different from each other, with the exception of carrot and potato. For a_w,_ the values after dehydration ranged from 0.221 ± 0.025 (for potato) to 0.262 ± 0.050 (for bell pepper) and were not significantly different. Compared to their fresh counterparts, all a_w_ and pH values for the dehydrated vegetables were significantly lower.

The moisture contents of the fresh, dehydrated, and rehydrated vegetables were also measured in this study and are presented in Table 2 and Figures S1 and S2. The moisture contents of the fresh vegetables ranged from 78.00 ± 0.15 (for potato) to 93.69 ± 1.28% (for bell pepper); all values were significantly different. The moisture contents for all vegetables significantly decreased after dehydration and ranged from 4.50 ± 0.68 (for potato) to 15.58 ± 0.94% (for onion). The moisture contents of both onion and bell pepper were significantly higher than the other three vegetables, whereas the moisture content of potato was significantly lower than the four other vegetables.

After 24 h of rehydration at either 5 or 25 °C, the moisture contents of all the dehydrated vegetables significantly increased. The rehydrated moisture contents ranged from 68.00 ± 4.39 (for potato rehydrated at 5 °C) to 94.19 ± 0.91% (for onion rehydrated at 25 °C). When comparing the differences between the two rehydration temperatures, the moisture contents after 24 h were not significantly different for all vegetables, with the exception of corn and bell pepper. Rehydration at 25 °C resulted in significantly higher moisture contents of both corn and bell pepper (83.36 ± 1.13 and 92.08 ± 1.41%, respectively) compared to 5 °C (79.39 ± 0.92 and 90.38 ± 0.26%, respectively). Not all vegetables rehydrated to the same moisture content levels as their fresh counterparts. Most notably, the moisture contents of potato after rehydration at 5 and 25 °C (68.00 ± 4.39 and 68.42 ± 4.27%, respectively) were significantly lower than the moisture content when fresh (78.00 ± 0.15%).

### 3.2. Survival of L. monocytogenes and S. enterica on Dehydrated Vegetables during Rehydration

The populations of *L. monocytogenes* and *S. enterica* on the vegetables prior to and after rehydration are depicted in Table 3 and Table 4, respectively. Prior to rehydration, the population of *L. monocytogenes* on the dehydrated vegetables after inoculation and drying for 24 h ranged from 1.99 ± 0.18 (on corn) to 2.81 ± 0.17 log CFU/g (on bell pepper). The pathogen population on corn was significantly lower than on carrot, bell pepper, or potato. Drying of the vegetables for 24 h resulted in an approximate 1.19–2.01 log CFU/g reduction of *L. monocytogenes*. After rehydration at 5 °C for 24 h, *L. monocytogenes* was below the limit of enumeration (1.70 log CFU/g) on all vegetables with the exception of potato, where the population was 1.85 ± 0.16 log CFU/g. After rehydration at 25 °C for 24 h, the *L. monocytogenes* populations ranged from 2.28 ± 0.20 (on corn) to 3.42 ± 0.35 log CFU/g (on potato). Similar to the initial populations, the levels on corn were significantly lower than on carrot, bell pepper, or potato.

Prior to rehydration, the population of *S. enterica* on the dehydrated vegetables after inoculation and drying for 24 h ranged from 1.82 ± 0.51 (on carrot) to 2.83 ± 0.09 log CFU/g (on potato). The pathogen population on carrot was significantly lower than on all the other vegetables. Drying of the vegetables for 24 h resulted in an approximate 1.17–2.18 log CFU/g reduction of *S. enterica*. After rehydration at 5 °C for 24 h, *S. enterica* was below the limit of enumeration on all vegetables with the exception of onion, where the population was 1.99 ± 0.15 log CFU/g. After rehydration at 25 °C for 24 h, the *S. enterica* populations ranged from 2.32 ± 0.50 (on onion) to 6.25 ± 0.10 log CFU/g (on potato). The pathogen populations on onion and corn were significantly lower than on the other three vegetables. Compared to *L. monocytogenes*, the *S. enterica* populations on the vegetables after rehydration at 25 °C were all significantly higher; the only exception was on onion, where the two pathogen populations were not significantly different.

### 3.3. Growth Kinetics of L. monocytogenes and S. enterica on Rehydrated Vegetables during Storage

**Figure 1:**
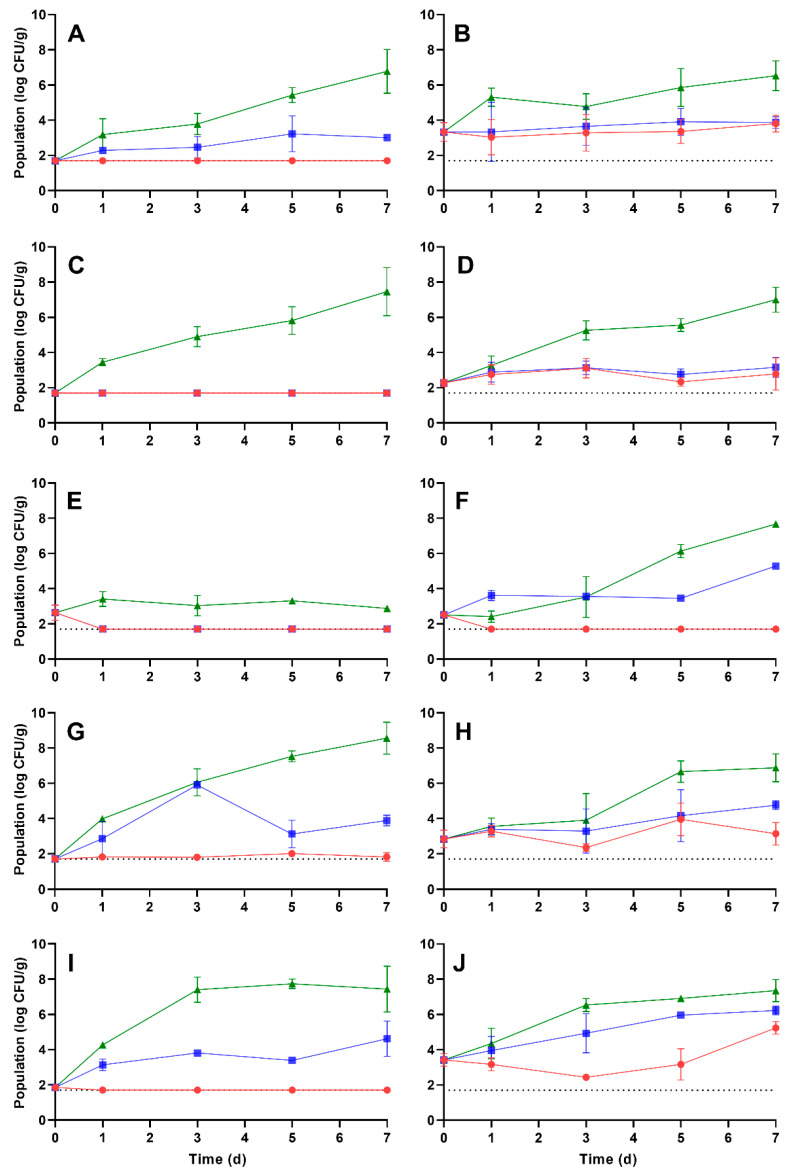
Population dynamics of *L. monocytogenes* on rehydrated carrot (**A**,**B**), corn (**C**,**D**), onion (**E**,**F**), pepper (**G**,**H**), and potato (**I**,**J**) during storage at 5 (red circles), 10 (blue squares), or 25 °C (green triangles) for 7 days. Vegetables were rehydrated at either 5 (**A**,**C**,**E**,**G**,**I**) or 25 °C (**B**,**D**,**F**,**H**,**J**). Data are mean values ± standard deviation (*n* = 9).

**Figure 2:**
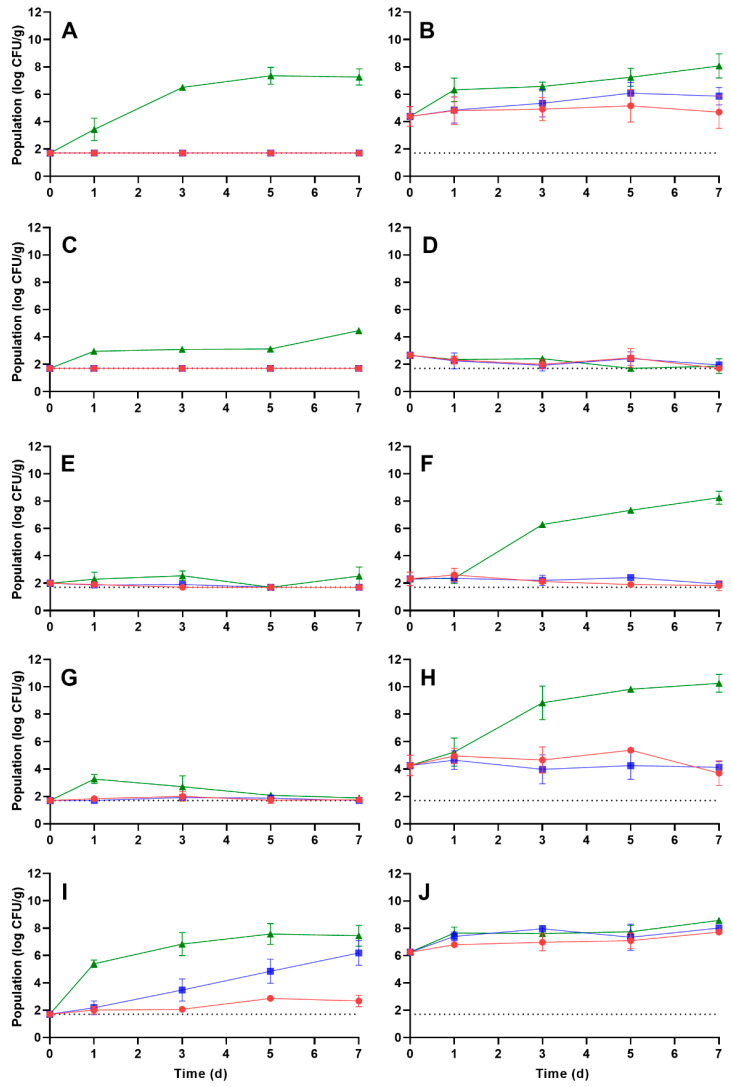
Population dynamics of *S. enterica* on rehydrated carrot (**A**,**B**), corn (**C**,**D**), onion (**E**,**F**), pepper (**G**,**H**), and potato (**I**,**J**) during storage at 5 (red circles), 10 (blue squares), or 25 °C (green triangles) for 7 days. Vegetables were rehydrated at either 5 (**A**,**C**,**E**,**G**,**I**) or 25 °C (**B**,**D**,**F**,**H**,**J**). Data are mean values ± standard deviation (*n* = 9).

After rehydration at either 5 or 25 °C, the vegetables were stored at 5, 10, or 25 °C for 7 d. The population dynamics of *L. monocytogenes* and *S. enterica* on the rehydrated vegetables during storage are presented in Figure 1 and Figure 2, respectively. Additionally, the growth rates, calculated times for a 1 log CFU/g increase in population, and the *L. monocytogenes* and *S. enterica* populations at the end of the 7 d storage period are depicted in Table 5 and Table 6, respectively. In general, storage at 25 °C resulted in the highest growth rates and populations after 7 d storage for both pathogens. For *L. monocytogenes*, no significant proliferation was observed during storage at 5 °C on carrots, corn, onion, or potato when rehydrated at 5 °C or on onion when rehydrated at 25 °C. At 10 °C storage, the *L. monocytogenes* populations increased significantly on pepper and potato regardless of the rehydration temperature; populations after 7 d were 3.89 ± 0.30 and 4.75 ± 0.25 (on pepper) and 4.62 ± 1.00 and 6.23 ± 0.24 log CFU/g (on potato) when rehydrated at 5 and 25 °C, respectively. The pathogen also increased significantly in population on onion at 10 °C when rehydrated at 25 °C, with a population of 5.29 ± 0.06 log CFU/g after 7 d storage; no significant population increase was observed when onion was rehydrated at 5 °C and stored at 10 °C. *L. monocytogenes* increased significantly in population on all vegetables stored at 25 °C, regardless of the rehydration temperature, with one exception: no proliferation was observed on onions rehydrated at 5 °C. With the exception of onion, the population of *L. monocytogenes* on the vegetables after 7 d storage at 25 °C ranged from 6.53 ± 0.84 (on carrots rehydrated at 25 °C) to 8.56 ± 0.91 log CFU/g (on pepper rehydrated at 5 °C).

The highest growth rate of *L. monocytogenes* during storage at 5 °C was observed on potato rehydrated at 25 °C (0.08 ± 0.08 log CFU/g/d), with a 1 log CFU/g increase in 12.50 d. At 10 °C, the highest growth rate was observed on potato rehydrated at 5 °C (1.35 ± 0.79 log CFU/g/d) where the time for a 1 log CFU/g increase was 1.74 d. At 25 °C storage, the lowest growth rate of *L. monocytogenes* (not including onion rehydrated at 5 °C) was observed on carrot rehydrated at 25 °C (0.39 ± 0.06 log CFU/g/d) resulting in a 1 log CFU/g increase in 2.56 d. The highest growth rate at 25 °C storage was observed on potato rehydrated at 5 °C (2.37 ± 0.61 log CFU/g/d) with an increase of 1 log CFU/g in only 0.42 d (or 10.08 h).

For *S. enterica*, no significant proliferation was observed on the vegetables during storage at 5 °C, with the exception of potato rehydrated at 25 °C, where the ending population after 7 d storage was 7.72 ± 0.13 log CFU/g (an increase of approximately 1.47 log CFU/g). At 10 °C, *S. enterica* significantly proliferated on only potato and carrot; populations after 7 d were 6.18 ± 0.90 and 8.02 ± 0.17 log CFU/g on potato when rehydrated at 5 and 25 °C, respectively, and 0.21 ± 0.06 log CFU/g on carrot when rehydrated at 25 °C. At 25 °C, *S. enterica* did not significantly grow on onions or peppers rehydrated at 5 °C or on corn rehydrated at 25 °C. When rehydrated at 5 °C and stored at 25 °C, the highest *S. enterica* populations after 7 d were observed on carrot and potato (7.26 ± 0.59 and 7.44 ± 0.76 log CFU/g, respectively). When rehydrated at 25 °C and stored at 25 °C, the highest population was observed after 7 d on pepper (10.25 ± 0.06 log CFU/g). This was also the highest population attained by either pathogen after 7 d at 25 °C.

In comparison to *L. monocytogenes*, many of the growth rates of *S. enterica* during storage on the vegetables were negative. These negative rates were observed at all storage temperatures on corn rehydrated at 25 °C, on onion stored at 5 and 10 °C at both rehydration temperatures, and on pepper rehydrated at 5 °C and stored at 5 and 25 °C and rehydrated at 25 °C and stored at 5 and 10 °C. At 5 °C, the highest growth rate of *S. enterica* was observed on potato rehydrated at 25 °C (0.16 ± 0.03 log CFU/g/d) resulting in a 1 log CFU/g increase in 6.65 d. At 10 °C, the highest growth rate was observed on potato rehydrated at 5 °C (0.67 ± 0.06 log CFU/g/d) with a 1 log CFU/g increase in only 1.49 d. During storage at 25 °C, the highest growth rate of *S. enterica* was observed on carrot rehydrated at 5 °C (1.63 ± 0.18 log CFU/g/d) resulting in a 1 log CFU/g increase in only 0.61 d (or 14.64 h).

## 4. Discussion

This study examined the population dynamics of two foodborne pathogens, *L. monocytogenes* and *S. enterica*, on dehydrated vegetables during rehydration and subsequent storage. Compared to their fresh state, dehydrated vegetables are a convenient option for retail establishments, restaurants, and consumers, as they have long shelf lives, are lightweight and take up minimal space when stored, and they retain much of their nutritional values [32]. Dehydrated vegetables are also versatile and can be consumed alone once rehydrated or incorporated into other foods. However, once dehydrated vegetables are rehydrated, the high a_w_ could provide an environment suitable for the proliferation of foodborne pathogens. Since rehydrated vegetables may be stored for hours or days prior to consumption, this study evaluated the need for time and temperature control for safety for these food commodities.

Two different rehydration temperatures were employed in this study, 5 and 25 °C. Whereas no published studies have evaluated the survival of foodborne pathogens during the rehydration of dehydrated vegetables, studies have examined the rehydration kinetics of different vegetables, including those used in this study [28,30]. One of these studies used high temperatures ranging from 40 to 80 °C to rehydrate myriad vegetables, including carrot, corn, onion, pepper, and potato, with an interest in examining the rehydration kinetics, equilibrium moisture contents, and quality characteristics [28]. The authors determined that the water temperature used for rehydration influenced the rehydration rate, as higher temperatures resulted in higher rehydration rates and thus lower times to reach equilibrium moisture. While high temperatures may be used to rehydrate dehydrated vegetables, especially if they are incorporated into hot soups or stews, retail establishments and consumers may also rehydrate these products in water at ambient temperature or in the refrigerator when guides or instructions do not include specific temperatures for rehydrating dehydrated vegetables.

This study determined that rehydration of dehydrated vegetables at 5 °C for 24 h resulted in a decrease in *L. monocytogenes* and *S. enterica* populations, whereas populations of both pathogens increased on carrot, corn, and potato and of *S. enterica* on bell pepper when rehydration occurred at 25 °C. Compared to *L. monocytogenes*, higher population increases were observed for *S. enterica* during rehydration at 25 °C; the highest increases were observed on potato (3.42 log CFU/g) and carrot (2.56 log CFU/g). Compared to the other vegetables used in this study, potato and carrot are both root vegetables and contain the highest carbohydrate contents (16 and 10%, respectively) [33]. The higher temperature of 25 °C coupled with the nutrient contents of these two root vegetables may have played a role in the proliferation of *S. enterica* during rehydration. Interestingly, neither pathogen grew on onion and only *L. monocytogenes* proliferated on corn during rehydration. Onions are known to have antimicrobial properties [34], which may have resulted in extended lag phases during adaptation to the environment, thereby hindering the proliferation of both pathogens.

Once rehydrated, vegetables may be stored for hours or days prior to use. This study examined *L. monocytogenes* and *S. enterica* survival and growth on rehydrated vegetables at three different storage temperatures: 5 °C (refrigeration), 10 °C (temperature abuse), and 25 °C (ambient). Overall, both pathogens survived on all vegetables during storage at 5 °C regardless of the rehydration temperature; the fastest 1 log CFU/g population increases were observed by both pathogens on potato rehydrated at 25 °C (12.50 and 6.65 days for *L. monocytogenes* and *S. enterica*, respectively). Similar growth rates of *L. monocytogenes* have been observed for freshly chopped broccoli and cauliflower stored at 4 °C (growth rates of 0.05–0.10 log CFU/g/d with extrapolated 1 log CFU/g increases in 10.00–20.00 days) [35]. However, higher growth rates of *L. monocytogenes* have also been observed on freshly chopped red and green bell pepper, cucumber, and avocado pulp stored at 5 °C (growth rates of 0.016–0.071 log CFU/g/h with 1 log CFU/g increases in 14.19–63.85 h (or 0.38–1.69 days) [36]. It is possible that the process of rehydration deterred the growth of the pathogens on the vegetables stored at 5 °C, and that growth rates may be higher when inoculated directly onto freshly chopped matrices.

During storage at the temperature abuse condition of 10 °C, potato rehydrated at either 5 or 25 °C supported the proliferation of both pathogens, while bell pepper rehydrated at either 5 or 25 °C also supported the growth of *L. monocytogenes*. On potato stored at 10 °C, the fastest 1 log CFU/g population increases were observed for both pathogens when rehydration occurred at 5 °C (1.74 and 1.49 days for *L. monocytogenes* and *S. enterica*, respectively) as opposed to 25 °C (2.33 and 11.11 days, respectively). When rehydrated at 25 °C, the native microbiota of the potato may have also proliferated, inducing competition for nutrients by either pathogen when subsequently stored at 10 °C. These results are comparable to the literature, as *L. monocytogenes* was observed to increase in population by approximately 2 log CFU/g on fresh potato tuber slices stored at 8 °C for 12 days [37]. On bell pepper stored at 10 °C, 1 log CFU/g population increases of *L. monocytogenes* occurred after 0.83 and 4.17 days when rehydration occurred at 5 or 25 °C, respectively. Similarly, with 5 °C storage, higher growth rates have been documented on freshly chopped green bell pepper stored at 10 °C, where *L. monocytogenes* increased 1 log CFU/g in 51.12–53.92 h (or 0.45–0.47 days) [36].

At the ambient storage condition of 25 °C, the growth rates of both *L. monocytogenes* and *S. enterica* on carrot, corn, and potato were always higher when rehydration occurred at 5 °C, whereas growth rates were always higher on onion and pepper when rehydration occurred at 25 °C. One of the most drastic differences in the two rehydration temperatures was observed with onions, where both pathogens did not proliferate during storage at 25 °C on onions rehydrated at 5 °C, but when rehydrated at 25 °C, 1 log increases occurred in 3.35 and 0.74 days for *L. monocytogenes* and *S. enterica*, respectively. These results suggest that the proliferation of both pathogens on rehydrated vegetables at ambient temperature is both rehydration temperature and matrix dependent. Storage at 4 °C, compared to 10 or 25 °C, has been shown to better preserve enzymes and other antimicrobial compounds in onions [38]. It is likely that rehydration of the onions in this study at 5 °C aided in the preservation of these compounds, resulting in an environment which did not support the proliferation of either pathogen during subsequent storage at 25 °C.

Prior to dehydration, it is recommended to blanch certain vegetables to inactivate enzymes, preserve color and flavor, and reduce the microbial population [1,39]. For example, blanching is recommended for potatoes to inactivate enzymes involved in browning. However, some exceptions include onions, peppers, mushrooms, and tomatoes, because blanching results in loss of flavor and color [39]; it is also not recommended to blanch onions or bell peppers prior to freezing [40]. For consistency, this study did not utilize blanching prior to dehydration of the vegetables. Since blanching was not used, the vegetables used in this study retained their native microbiota, which may have impacted the results. Future studies could examine the fate of both *L. monocytogenes* and *S. enterica* during rehydration of blanched and dehydrated vegetables. It is possible that growth rates of both pathogens would be higher during rehydration and subsequent storage as no competition for nutrients would be required.

## 5. Conclusions

This study determined that the survival and proliferation of both *L. monocytogenes* and *S. enterica* on dehydrated vegetables during rehydration and storage was dependent on many variables, including the rehydration temperature, the temperature of storage, and the matrix characteristics. While both pathogens survived during rehydration and subsequent storage on all the dehydrated vegetables examined in this study, lower growth rates were generally observed when the vegetables were rehydrated at 5 °C and then stored at 5 °C. These results highlight the importance of holding rehydrated vegetables at refrigeration temperatures to hinder pathogen proliferation. Data for this study can be used to inform regulatory decisions surrounding time and temperature control for safety for these food products.

## Figures and Tables

**Table 1:** pH and water activity (a_w_) values of the fresh and dehydrated vegetables.

Vegetable	Fresh ^1^		Dehydrated ^2^	
	pH	a_w_	pH	a_w_
Carrot	7.00 ± 0.10 ^aA^	0.956 ± 0.002 ^aA^	6.50 ± 0.22 ^aB^	0.250 ± 0.070 ^aB^
Corn	6.90 ± 0.14 ^aA^	0.951 ± 0.015 ^aA^	6.77 ± 0.02 ^bB^	0.261 ± 0.008 ^aB^
Onion	6.79 ± 0.57 ^aA^	0.952 ± 0.014 ^aA^	5.73 ± 0.07 ^cB^	0.244 ± 0.011 ^aB^
Pepper	6.15 ± 0.06 ^bA^	0.956 ± 0.010 ^aA^	6.01 ± 0.04 ^dB^	0.262 ± 0.050 ^aB^
Potato	6.77 ± 0.09 ^aA^	0.960 ± 0.008 ^aA^	6.42 ± 0.04 ^aB^	0.221 ± 0.025 ^aB^

^1^, freshly cut. ^2^, after 24 h dehydration at 60 °C. Different lowercase letters indicate significant difference between pH or a_w_ values of vegetables in the same state (columns). Different uppercase letters indicate difference between pH or a_w_ values of vegetables between states (rows).

**Table 2:** The moisture contents of the fresh, dehydrated, and rehydrated vegetables.

Vegetable	Moisture (%)			
	Fresh ^1^	Dehydrated ^2^	Rehydrated ^3^	
			5 °C	25 °C
Carrot	88.61 ± 1.00 ^aA^	8.20 ± 1.13 ^aB^	88.52 ± 1.59 ^aA^	88.16 ± 1.46 ^aA^
Corn	82.25 ± 0.22 ^bA^	7.27 ± 0.84 ^aB^	79.39 ± 0.92 ^bC^	83.36 ± 1.13 ^bD^
Onion	91.90 ± 1.45 ^cA^	15.58 ± 0.94 ^bB^	92.94 ± 1.40 ^cAC^	94.19 ± 0.91 ^cC^
Pepper	93.69 ± 1.28 ^dA^	11.40 ± 1.83 ^cB^	90.38 ± 0.26 ^acC^	92.08 ± 1.41 ^cA^
Potato	78.00 ± 0.15 ^eA^	4.50 ± 0.68 ^dB^	68.00 ± 4.39 ^dC^	68.42 ± 4.27 ^dC^

^1^, freshly cut. ^2^, after 24 h dehydration at 60 °C. ^3^, after 24 h rehydration at either 5 or 25 °C (water and air temperature). Different lowercase letters indicate significant difference between the moisture contents of vegetables in the same state (columns). Different uppercase letters indicate difference between the moisture contents of vegetables between states (rows).

**Table 3:** *L. monocytogenes* population dynamics on dehydrated vegetables prior to and after 24 h rehydration at 5 or 25 °C.

Vegetable	Initial Population ^1^ (log CFU/g ± SD ^2^)	Population after Rehydration ^3^ (log CFU/g ± SD)	
		5 °C	25 °C
Carrot	2.70 ± 0.20 ^aA^	<1.70	3.34 ± 0.54 ^aB^
Corn	1.99 ± 0.18 ^bA^	<1.70	2.28 ± 0.20 ^bB^
Onion	2.50 ± 0.90 ^abA^	<1.70	2.51 ± 0.11 ^bcA^
Pepper	2.80 ± 0.07 ^aA^	<1.70	2.84 ± 0.50 ^acA^
Potato	2.62 ± 0.19 ^aA^	1.85 ± 0.16 ^B^	3.42 ± 0.35 ^aC^

^1^, initial population after inoculation and 24 h drying, prior to rehydration. ^2^, standard deviation. ^3^, after 24 h rehydration at either 5 or 25 °C (water and air temperature). Different lowercase letters indicate significant difference between the populations on vegetables in the same state (columns). Different uppercase letters indicate difference between the populations on vegetables between states (rows).

**Table 4:** *S. enterica* population dynamics on dehydrated plant foods prior to and after 24 h rehydration at 5 or 25 °C.

Vegetable	Initial Population ^1^ (log CFU/g ± SD ^2^)	Population after Rehydration ^3^ (log CFU/g ± SD)	
		5 °C	25 °C
Carrot	1.82 ± 0.51 ^aA^	<1.70	4.38 ± 0.73 ^aB^
Corn	2.54 ± 0.23 ^bA^	<1.70	2.66 ± 0.13 ^bA^
Onion	2.46 ± 0.40 ^bA^	1.99 ± 0.15 ^B^	2.32 ± 0.50 b^AB^
Pepper	2.82 ± 0.08 ^bA^	<1.70	4.27 ± 0.75 ^aB^
Potato	2.83 ± 0.09 ^bA^	<1.70	6.25 ± 0.10 ^cB^

^1^, initial population after inoculation and 24 h drying, prior to rehydration. ^2^, standard deviation. ^3^, after 24 h rehydration at either 5 or 25 °C (water and air temperature). Different lowercase letters indicate significant difference between the populations on vegetables in the same state (columns). Different uppercase letters indicate difference between the populations on vegetables between states (rows).

**Table 5:** Growth kinetics of *L. monocytogenes* on rehydrated vegetables during subsequent storage at 5, 10, or 25 °C for 7 days.

Vegetable	Rehydration Temperature (°C) ^1^	Storage Temperature (°C)	Growth Rate (log CFU/g per d ± SE ^2^)	Time (d) to a 1 log CFU/g Increase ^4^	Population after 7 d Storage (log CFU/g ± SD ^3^)
Carrot	5	5	ND ^5^	NA ^6^	<1.70
		10	ND	NA	3.01 ± 0.18 ^a^
		25	0.63 ± 0.07 ^a^	1.59	6.78 ± 1.24 ^b^
	25	5	0.07 ± 0.05 ^b^	14.29	3.81 ± 0.48 ^c^
		10	0.10 ± 0.07 ^b^	10.00	3.88 ± 0.33 ^c^
		25	0.39 ± 0.06 ^c^	2.56	6.53 ± 0.84 ^b^
Corn	5	5	ND	NA	<1.70
		10	ND	NA	<1.70
		25	0.67 ± 0.06 ^a^	1.49	7.46 ± 1.37 ^a^
	25	5	0.03 ± 0.04 ^b^	33.33	2.79 ± 0.91 ^b^
		10	0.51 ± 0.22 ^a^	1.96	3.16 ± 0.56 ^b^
		25	0.63 ± 0.04 ^a^	1.59	7.00 ± 0.71 ^a^
Onion	5	5	ND	NA	<1.70
		10	ND	NA	<1.70
		25	0.04 ± 0.05 ^a^	25	2.88 ± 0.08 ^a^
	25	5	ND	NA	<1.70
		10	0.32 ± 0.05 ^b^	3.13	5.29 ± 0.06 ^b^
		25	1.05 ± 0.15 ^c^	2.80 ^7^	7.67 ± 0.07 ^c^
Pepper	5	5	0.02 ± 0.02 ^a^	50.00	1.82 ± 0.24 ^a^
		10	1.20 ± 1.18 ^b^	0.83	3.89 ± 0.30 ^bc^
		25	1.02 ± 0.11 ^b^	0.98	8.56 ± 0.91 ^d^
	25	5	0.05 ± 0.04 ^a^	20.00	3.14 ± 0.64 ^b^
		10	0.24 ± 0.06 ^b^	4.17	4.75 ± 0.25 ^c^
		25	1.66 ± 0.60 ^b^	3.35 ^8^	6.88 ± 0.79 ^d^
Potato	5	5	ND	NA	<1.70
		10	1.35 ± 0.79 ^a^	1.74	4.62 ± 1.00 ^a^
		25	2.37 ± 0.61 ^a^	0.42	7.44 ± 1.30 ^b^
	25	5	0.08 ± 0.08 ^b^	12.50	5.24 ± 0.35 ^a^
		10	0.43 ± 0.04 ^c^	2.33	6.23 ± 0.24 ^b^
		25	1.07 ± 0.11 ^a^	0.93	7.35 ± 0.63 ^b^

^1^, water and air temperature; ^2^, standard error; ^3^, standard deviation; ^4^, calculated based on growth rate; ^5^, not determined; ^6^, not applicable; ^7^, DMFit predicted a lag phase of 1.85 ± 0.61 d, which was taken into consideration when calculating time for a 1 log CFU/g increase; ^8^, DMFit predicted a lag phase of 2.75 ± 0.58 d, which was taken into consideration when calculating time for a 1 log CFU/g increase. Different lowercase letters indicate significant difference between the growth rates or populations at different temperatures on the same vegetable.

**Table 6:** Growth kinetics of *S. enterica* on rehydrated vegetables during subsequent storage at 5, 10, or 25 °C for 7 days.

Vegetable	Rehydration Temperature (°C) ^1^	Storage Temperature (°C)	Growth Rate (log CFU/g per d ± SE ^2^)	Time (d) to a 1 log CFU/g Increase ^4^	Population after 7 d Storage (log CFU/g ± SD ^3^)
Carrot	5	5	0.01 ± 0.03 ^a^	100	<1.70
		10	0.03 ± 0.30 ^a^	33.33	<1.70
		25	1.63 ± 0.18 ^b^	0.61	7.26 ± 0.59 ^a^
	25	5	0.05 ± 0.06 ^a^	20.00	4.69 ± 1.19 ^b^
		10	0.21 ± 0.06 ^c^	4.76	5.86 ± 0.64 ^b^
		25	0.44 ± 0.05 ^d^	2.27	8.07 ± 0.88 ^c^
Corn	5	5	ND ^5^	NA ^6^	<1.70
		10	ND	NA	<1.70
		25	0.31 ± 0.04 ^a^	3.22	4.47 ± 0.09 ^a^
	25	5	−0.10 ± 0.05 ^b^	NA	<1.70
		10	−0.06 ± 0.05 ^b^	NA	1.95 ± 0.07 ^b^
		25	−0.12 ± 0.04 ^b^	NA	1.88 ± 0.53 ^b^
Onion	5	5	−0.05 ± 0.02 ^a^	NA	<1.70
		10	−0.04 ± 0.02 ^a^	NA	<1.70
		25	0.04 ± 0.05 ^b^	25.00	2.53 ± 0.66 ^a^
	25	5	−0.09 ± 0.03 ^a^	NA	1.82 ± 0.36 ^a^
		10	−0.04 ± 0.05 ^a^	NA	1.93 ± 0.04 ^a^
		25	1.36 ± 0.35 ^c^	0.74 ^7^	8.25 ± 0.47 ^b^
Pepper	5	5	−0.02 ± 0.03 ^a^	NA	1.75 ± 0.18 ^a^
		10	0.01 ± 0.03 ^a^	100.00	<1.70
		25	−0.18 ± 0.08 ^b^	NA	1.89 ± 0.16 ^a^
	25	5	−0.04 ± 0.09 ^a^	NA	3.70 ± 0.91 ^b^
		10	−0.05 ± 0.08 ^a^	NA	4.13 ± 0.40 ^b^
		25	1.49 ± 0.24 ^c^	0.67	10.25 ± 0.65 ^c^
Potato	5	5	0.14 ± 0.03 ^a^	7.14	2.67 ± 0.42 ^a^
		10	0.67 ± 0.06 ^b^	1.49	6.18 ± 0.90 ^b^
		25	1.13 ± 0.24 ^c^	0.89	7.44 ± 0.76 ^bc^
	25	5	0.16 ± 0.03 ^a^	6.65	7.72 ± 0.13 ^c^
		10	0.09 ± 0.05 ^a^	11.11	8.02 ± 0.17 ^d^
		25	0.17 ± 0.04 ^a^	5.88	8.57 ± 0.17 ^e^

^1^, water and air temperature; ^2^, standard error; ^3^, standard deviation; ^4^, calculated based on growth rate; ^5^, not determined; ^6^, not applicable; ^7^, DMFit predicted a lag phase of 0.63 ± 0.89 d, which was taken into consideration when calculating time for a 1 log CFU/g increase. Different lowercase letters indicate significant difference between the growth rates at different temperatures for the same vegetable.

## Data Availability

All data is contained within the article.

## Supplementary Materials

The following supporting information can be downloaded at: https://www.mdpi.com/article/10.3390/foods12132561/s1, Figure S1: The moisture contents (%) of the vegetables during dehydration at 60°C for 24 h. Data are mean values ± standard deviation (*n* = 9); Figure S2: The moisture contents (%) of the vegetables during rehydration at (A) 5 or (B) 25°C for 24 h. Data are mean values ± standard deviation (*n* = 9).
