# Supplementary material for: Growth Kinetics of Listeria monocytogenes and Salmonella enterica on Dehydrated Vegetables during Rehydration and Subsequent Storage
Source: Foods. 2023 Jun 30;12(13):2561. doi: 10.3390/foods12132561 (PMC10341089; doi:10.3390/foods12132561)

Supplemental Figures

Figure S1. The moisture contents (%) of the vegetables during dehydration at 60°C for 24 h. Data are mean values ± standard deviation (n=9).

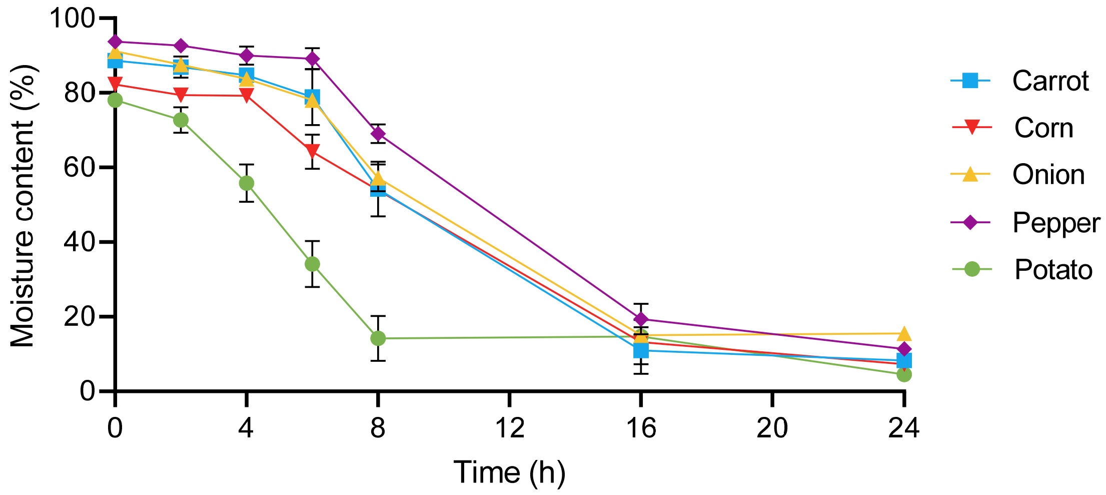

Figure S2. The moisture contents (%) of the vegetables during rehydration at A) 5 or B) 25°C for 24 h. Data are mean values ± standard deviation (n=9).

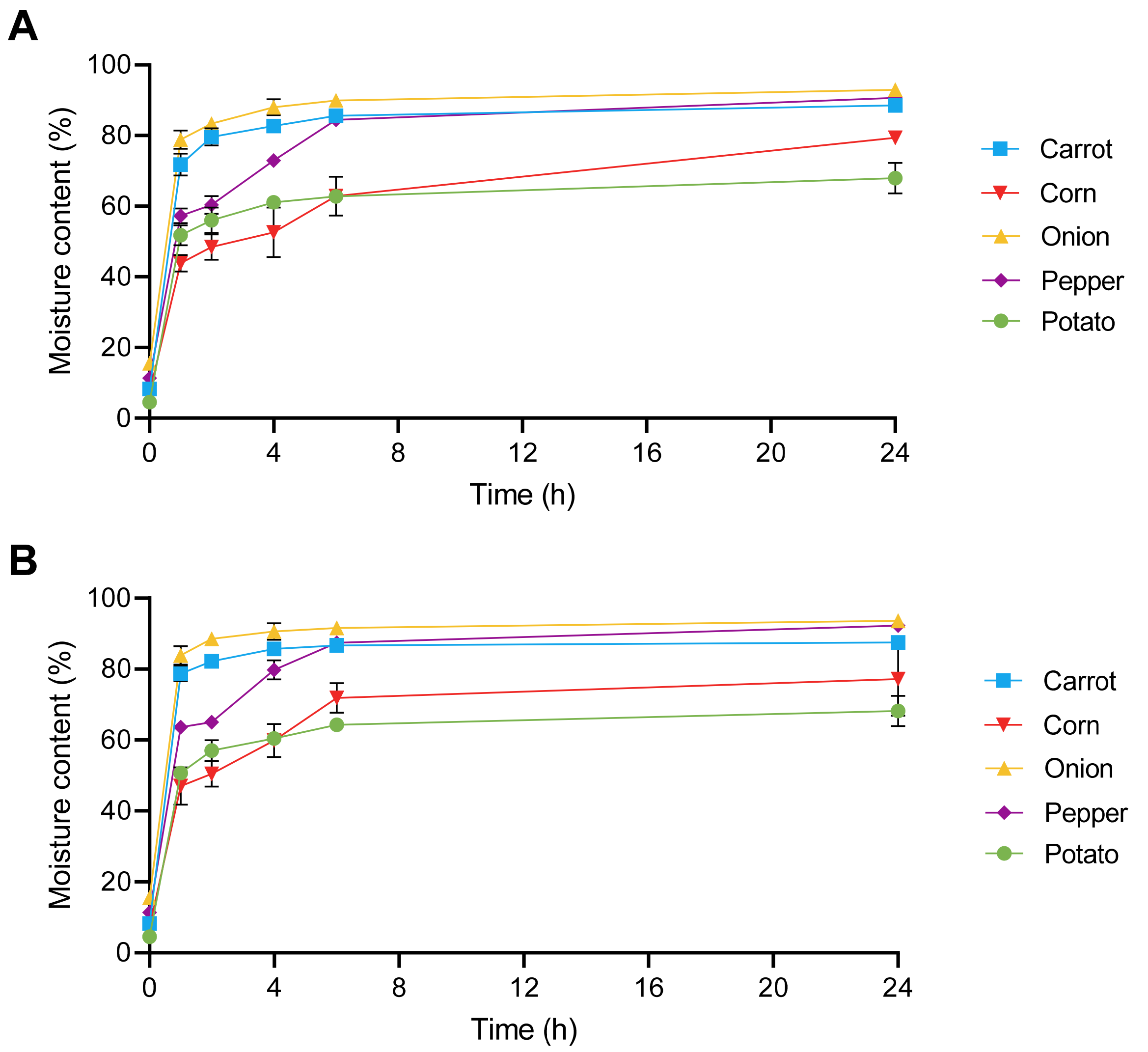

Supplement: Supplementary file 1 [file foods-12-02561-s001.zip › foods-2437842-supplementary.pdf]
